# Supplementary material for: HIF-Overexpression and Pro-Inflammatory Priming in Human Mesenchymal Stromal Cells Improves the Healing Properties of Extracellular Vesicles in Experimental Crohn’s Disease
Source: Int J Mol Sci. 2021 Oct 19;22(20):11269. doi: 10.3390/ijms222011269 (PMC8540690; doi:10.3390/ijms222011269)
Supplement: Supplementary file 1 [file ijms-22-11269-s001.zip › ijms-1409791-supplementary.pdf]

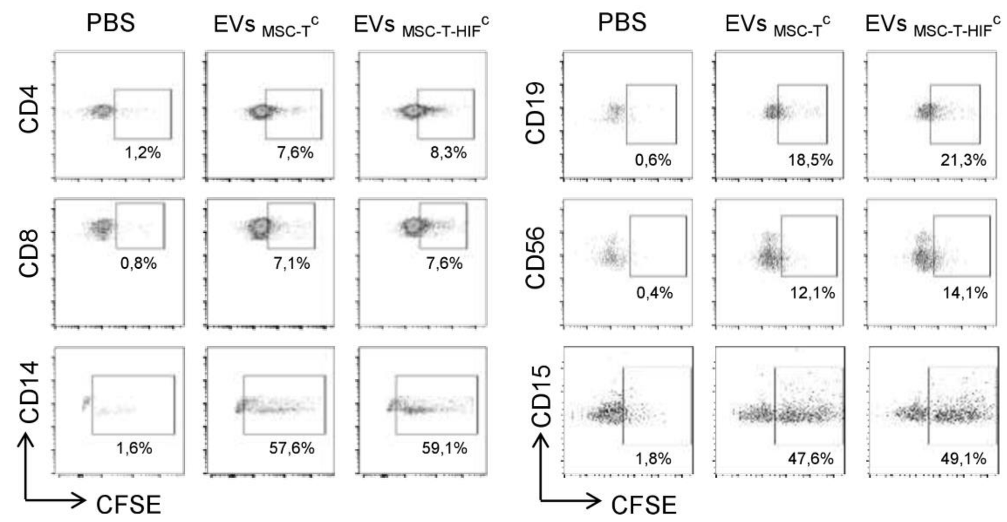

Supplementary Figure S1. Representative dot plots show the percentage of CFSE- labeled EV uptake by T-cells ($CD4^+$ and $CD8^+$), B-cells ($CD19^+$), monocytes ($CD14^+$), NK cells ($CD56^+$) and neutrophils ($CD15^+$).

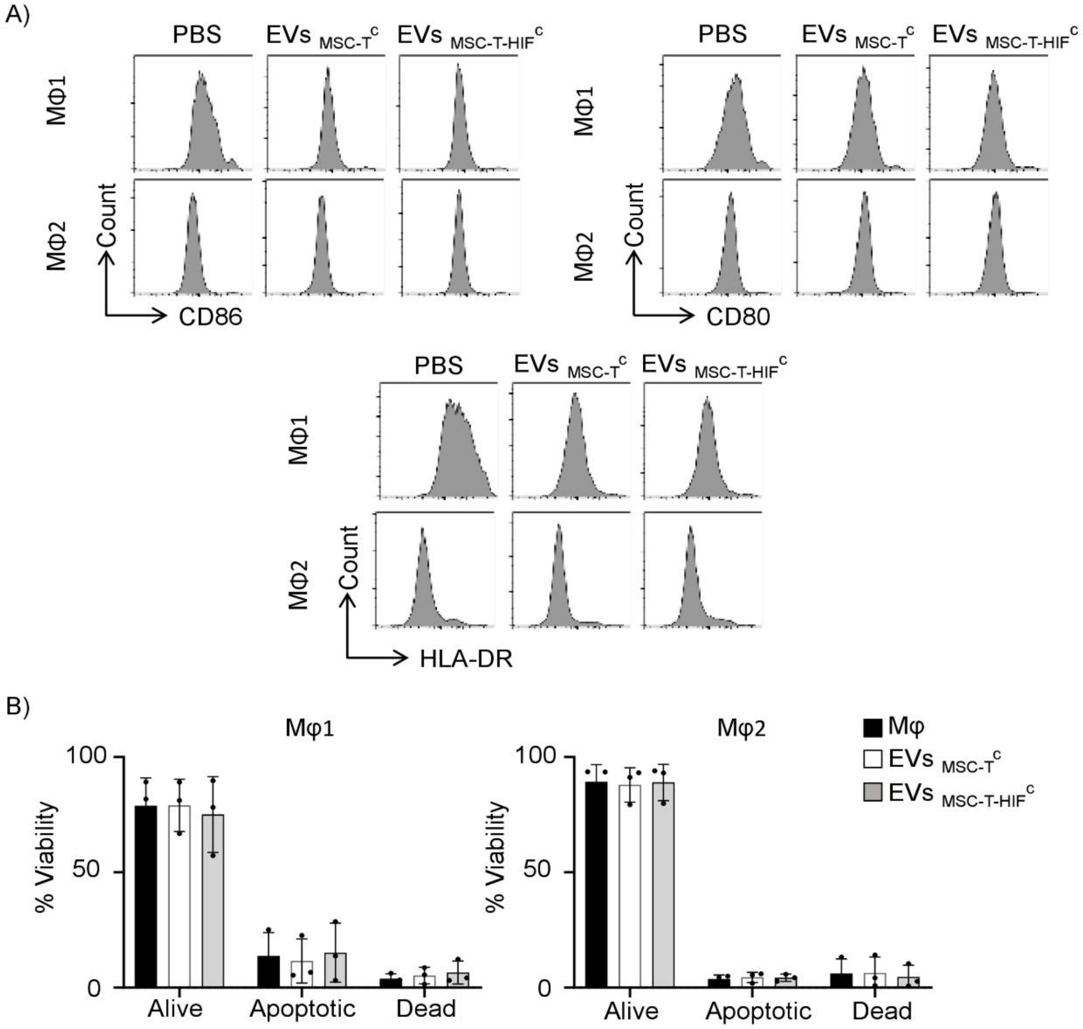

Supplementary Figure S2. $EV_{MSC\text{-}T}{}^{C}$ and $EV_{MSC\text{-}T\text{-}HIF}{}^{C}$ do not affect the viability of differentiating macrophages. (**A**) Quantification of annexin V-FITC and propidium iodide positive cells. Graphs show viable, apoptotic and dead macrophages after M1/M2 differentiation in presence or not of EVs. (**B**) Representative dot plots of CD86, CD80 and HLA-DR expression assessed by flow cytometry after LPS-activation.

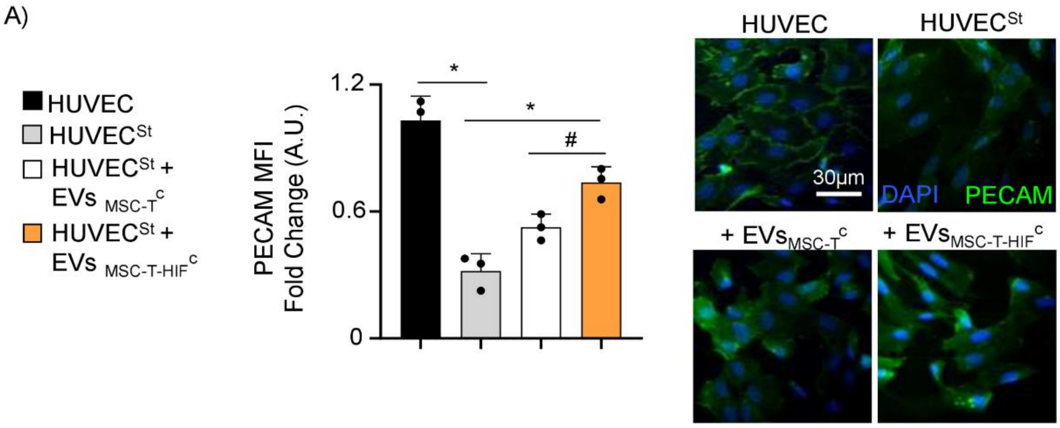

Supplementary Figure S3. Immunofluorescence of PECAM (CD31, green) and staining of nuclei (blue) to show distribution of PECAM in cell membrane. Scale bar: 30 µg. Bar graph shows quantification of green mean fluorescence intensity (MFI). Relative MFI was calculated by dividing all individual data by the MFI in unstimulated HUVECs. Graphs represent mean ± SD of fold change of three independent experiments. One-way ANOVA with Geisser-Greenhouse correction was used for statistical analysis.
